# Multivalent γ‐PGA‐Exendin‐4 Conjugates to Target Pancreatic β‐Cells

**DOI:** 10.1002/cbic.202200196

**Published:** 2022-07-13

**Authors:** Lorenzo Rossi, Krisztina Kerekes, Judit Kovács‐Kocsi, Zoltán Körhegyi, Magdolna Bodnár, Erika Fazekas, Eszter Prépost, Cataldo Pignatelli, Enrico Caneva, Francesco Nicotra, Laura Russo

**Affiliations:** ^1^ Department of Biotechnology ad Biosciences University of Milano-Bicocca Piazza della Scienza 2 20126 Milan Italy; ^2^ BBS Nanotechnology Ltd. Böszörményi út 212 4032 Debrecen Hungary; ^3^ UNITECH COSPECT Comprehensive Substances characterization via advanced sPECTtroscopy Via C. Golgi 19 20133 Milan Italy

**Keywords:** beta-cell targeting, diabetes, exendin-4, GLP-1R, pancreatic tumor, poly-gamma-glutamic acid

## Abstract

Targeting of glucagon‐like peptide 1 receptor (GLP‐1R), expressed on the surface of pancreatic β‐cells, is of great interest for the development of advanced therapies for diabetes and diagnostics for insulinoma. We report the conjugation of exendin‐4 (Ex‐4), an approved drug to treat type 2 diabetes, to poly‐γ‐glutamic acid (γ‐PGA) to obtain more stable and effective GLP‐1R ligands. Exendin‐4 modified at Lysine‐27 with PEG4‐maleimide was conjugated to γ‐PGA functionalized with furan, in different molar ratios, exploiting a chemoselective Diels‐Alder cycloaddition. The γ‐PGA presenting the highest number of conjugated Ex‐4 molecules (average 120 per polymeric chain) showed a double affinity towards GLP‐1R with respect to exendin per se, paving the way to improved therapeutic and diagnostic applications.

## Graphical abstract

**Exendin‐4‐γ‐polyglutamic acid** conjugates have been generated by efficient Diels‐Alder click reaction. The highly functionalized conjugate (average 106 Exendin‐4 molecules/γ‐Polyglutamic acid) showed affinity towards GLP1 receptor higher with respect to Enendin‐4 a labile peptide approved for type 2 diabetes treatment.

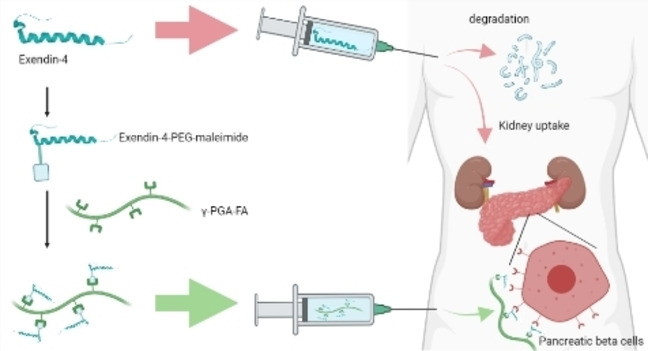

## Introduction

Type 2 diabetes is an unresolved pathology, related to genetic and environmental factors, which gradually decline the pancreatic islet functionality, finally culminating in a pathological phenomenon defined as hyperglycemia.^[1]^ Pancreatic β‐cells take on a key role in the so‐called glucose homeostasis by secreting insulin and amylin.^[2]^ A wide range of pharmacologic treatments have been developed, but none of them is considered the ultimate solution.^[3]^ The classic target for diabetes 2 therapies is GLP‐1R, that stimulates insulin secretion upon interaction with its natural ligand, glucagon‐like peptide 1 (GLP‐1). GLP‐1R is highly expressed on pancreatic β‐cells, and in case of certain endocrine and neuroendocrine cancers, including insulinoma, the density of such receptor is even higher, representing a valuable molecular target.[^4^, ^5^] Its natural ligand GLP‐1 is secreted by intestine in response to nutrients intake,^[6]^ but is rapidly (<2 minutes) degraded by dipeptidyl peptidase‐IV (DPP‐IV), making the use of more stable agonists necessary for therapeutic purposes.[^7^, ^8^] Ex‐4 is a 39 amino acid peptide with a higher half‐life in plasma,^[9]^ capable of binding GLP‐1R selectively with picomolar activity. For this reason, it finds wide use as targeting probe for GLP‐1R,[^10^, ^11^] and represents a promising therapeutic approach for type 2 diabetes.^[12]^ Exenatide, the synthetic form of Ex‐4, has been approved by FDA in 2005.^[13]^ However, besides its longer half‐life and clear advantages, Ex‐4 still shares some issues with GLP‐1 and, as other therapeutic peptides, its clinical use is limited by the poor stability in the body, the high uptake by liver and kidneys,^[14]^ the rapid clearance by kidneys because of its low molecular weight,[^15^, ^16^, ^17^, ^18^, ^19^] and the side effects related to the administration route and dosage.[^20^, ^21^] Therefore, the development of Ex‐4 derivatives with improved stability and different administration routes is desirable to increase the translational potential.^[22]^ Recently, multiple strategies for the synthesis of enhanced GLP‐1R agonists have been considered and tested,^[23]^ including terminal region modification,^[24]^ cyclization,[^25^, ^26^, ^27^] PEGylation,[^28^, ^29^, ^30^, ^31^] stapling,^[32]^ glycosylation,[^33^, ^34^] conjugation with lipids,^[35]^ incorporation of non‐natural amino acids,[^36^, ^37^] but each of them can efficaciously address only one specific issue.^[18]^ Therefore, the design of more stable and potent GLP‐1 receptor agonists is still of great interest.

The conjugation of Ex‐4 with biodegradable FDA approved polymers in a multivalent fashion can represent an innovative, affordable and effective therapeutic solution. Therefore, the simultaneous exhibition of multiple GLP‐1R agonists may lead to a cluster effect, enhancing the relative affinity of each of them towards the target receptor.[^38^, ^39^, ^40^, ^41^] Moreover, the conjugation of peptides to macromolecules bigger than 60 kDa, of different nature, has been reported to reduce renal uptake and clearance,[^29^, ^42^, ^43^] while extending the half‐life in the organism.[^44^, ^45^, ^46^, ^47^]

Herein we present an efficient and chemoselective protocol for the conjugation of Ex‐4 to γ‐PGA. γ‐PGA exhibits multiple attractive properties, including absence of toxicity, biodegradability, edibility, and water solubility.^[48]^ Consequently γ‐PGA has found application in multiple fields such as the pharmaceutical, nutraceutical and agricultural industries.^[49]^ Medical uses include drug carrier,[^50^, ^51^] scaffold component for tissue engineering,^[52]^ surgical adhesives^[53]^ and MRI probes.^[54]^ The units of glutamic acid are polymerized via γ‐amide bonds, while his carboxylic groups offer attachment points along the chain for both targeting and contrast agents. γ‐PGA has already been used to inhibit kidney uptake of radiolabeled peptides, including Ex‐4, by co‐administration,^[55]^ even if the mechanism has not been fully clarified.^[16]^ Administration of a stable γ‐PGA based therapeutic or diagnostic will in principle dramatically decrease the kidney uptake, increasing its activity towards GLP‐1R.

A γ‐PGA‐Ex‐4 conjugate may represent the starting point for the development of novel therapeutic polymeric nanotools targeting β‐cells with high specificity. γ‐PGA, and therefore γ‐PGA‐Ex‐4 conjugate, is stable in aqueous solutions, requiring high temperatures and low pH values^[56]^ for degradation, but interestingly it will be hydrolyzed and metabolized by mammals.^[57]^

The generation of polymer conjugates for therapeutic purposes requires efficient, chemoselective and clean protocols usually defined click reactions. Their attractive features include very high yields (quantitative), modularity, absence of toxic byproducts.[^58^, ^59^, ^60^, ^61^] In addition to this, reactions such as Diels‐Alder cycloaddition,[^62^, ^63^] thiol Michael addition,[^64^, ^65^] or strain‐promoted azide‐alkyne reaction[^66^, ^67^] occur in mild biocompatible conditions,[^59^, ^68^, ^69^, ^70^] and have already been exploited to obtain molecular probes or therapeutic drug conjugates.

**Figure 1:**
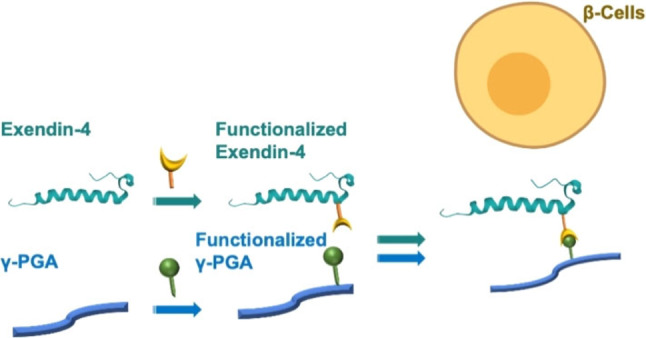
Representation of the modified Exendin‐4 conjugated to functionalized γ‐PGA, accomplishing the interaction with β‐TC6 cells.

We developed an efficient click chemistry protocol based on Diels‐Alder cycloaddition to conjugate Ex‐4 to γ‐PGA and evaluated the affinity of the γ‐PGA‐Ex4 conjugate towards GLP‐1 receptors of β‐TC6 cells (Figure 1).

## Results and Discussion

**Scheme 1:**
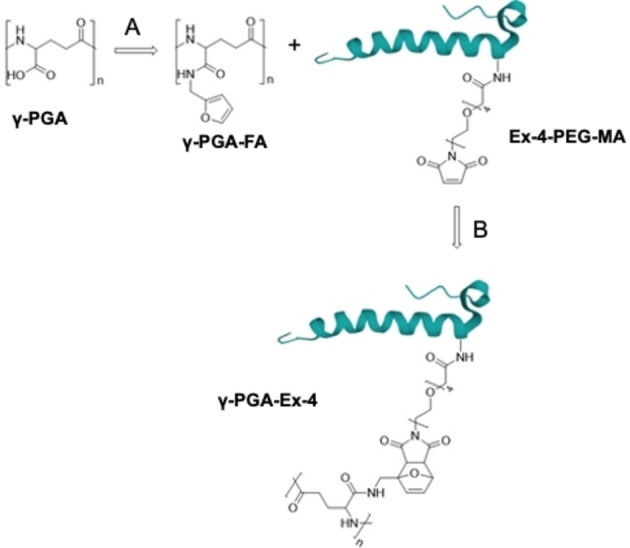
Chemical route of γ‐PGA‐Ex‐4 synthesis. A: MES 0.1 м pH=5.5, EDC, NHS, FA. B: PBS 0.01 м pH=7.5. Abbreviations: γ‐PGA: poly‐γ‐glutamic acid, γ‐PGA‐FA: poly‐γ‐glutamic acid‐furfurylamide, Ex‐4‐PEG‐MA: exendin‐4(Lysine27‐PEG4‐maleimide), γ‐PGA‐Ex‐4: poly‐γ‐glutamic acid ‐ exendin‐4 conjugate.

Diels‐Alder cycloaddition reaction was selected to conjugate of Ex‐4 to γ‐PGA (Scheme 1). To this purpose we exploited an Ex‐4 functionalized with a linker containing a maleimide group, acting as dienophile for a Diels‐Alder reaction. The derivatization site on lysine is a crucial aspect to be considered, since observations of crystal structure reveal that Ex‐4 interacts with GLP‐1R with multiple hydrophilic and hydrophobic residues.[^16^, ^71^] Nevertheless, literature reveals how different modifications on more positions have been successfully carried out and evaluated, including positions 1 (histidine),^[72]^ 12 (lysine, Lys),[^73^, ^74^] 14 (methionine),^[75]^ 27 (Lys),[^7^, ^76^] 39 (serine),^[77]^ and 40 (Lys).[^78^, ^79^, ^80^, ^81^, ^82^, ^83^] Lysins are the most exploited amino acid in Ex‐4 for bioconjugates synthesis, displaying a primary amine suitable for chemical derivatization. Based on literature information, functionalization of Ex‐4 at Lys‐27 do not compromise the interaction with GLP‐1R,^[35]^ but results in a slower internalization,^[81]^ which leads to an even more sustained release. Therefore, we employed an Ex‐4 functionalized at Lys‐27 with a short PEG spacer (4 glycol units) terminating with maleimide. γ‐PGA was complementarily functionalized with a furan as diene counterpart. To this purpose, the carboxylic group of γ‐PGA was condensed with furfurylamine (FA) using 1‐ethyl‐3‐(3‐dimethylaminopropyl)carbodiimide (EDC) and *N*‐hydroxysuccinimide (NHS) as condensing agents.

## Synthesis and characterization of γ‐PGA‐FA

Different degrees of functionalization (%DoF), 2.1 %, 8.5 % and 16.8 %, were obtained adjusting the amount of FA and EDC/NHS (Table 1).

**Table 1:** Equivalents and degree of functionalization for γ‐PGA‐FA‐**1**, γ‐PGA‐FA‐**2**, γ‐PGA‐FA‐**3**.

Sample	EDC:NHS:F [eq.]	%DoF
γ‐PGA‐FA‐1	0.5 : 0.5 : 0.25	2.1 ±0.4 %
γ‐PGA‐FA‐2	1 : 1 : 0.5	8.5 ±0.5 %
γ‐PGA‐FA‐3	2 : 2 : 2	16.8 ±0.1 %

The resulting polymers γ‐PGA‐FA‐**1**, γ‐PGA‐FA‐**2** and γ‐PGA‐FA‐**3** were characterized by NMR and FT‐IR analysis to verify the reproducibility and to determine the degree of functionalization.

The assignment of the peaks in NMR spectra was first carried out for γ‐PGA‐FA‐**1** by evaluation of chemical shifts, integrals and 2D experiments. In detail, the γ‐PGA protons correlations and the furan protons correlations were characterized via ^1^H‐^1^H COSY. The derivatization of γ‐PGA with furan was verified by DOSY, while the ^1^H‐^13^C long correlation showed the connection through an amide bond by the interaction between the CH_2_ of furan and C=O−NH protons. The assignment of the peaks of ^13^C spectra was confirmed by ^1^H‐^13^C HSQC. Based on this in‐depth analysis, the peaks of γ‐PGA‐FA‐**2** and γ‐PGA‐FA‐**3** were also assigned (details in the Supporting Information).

**Figure 2:**
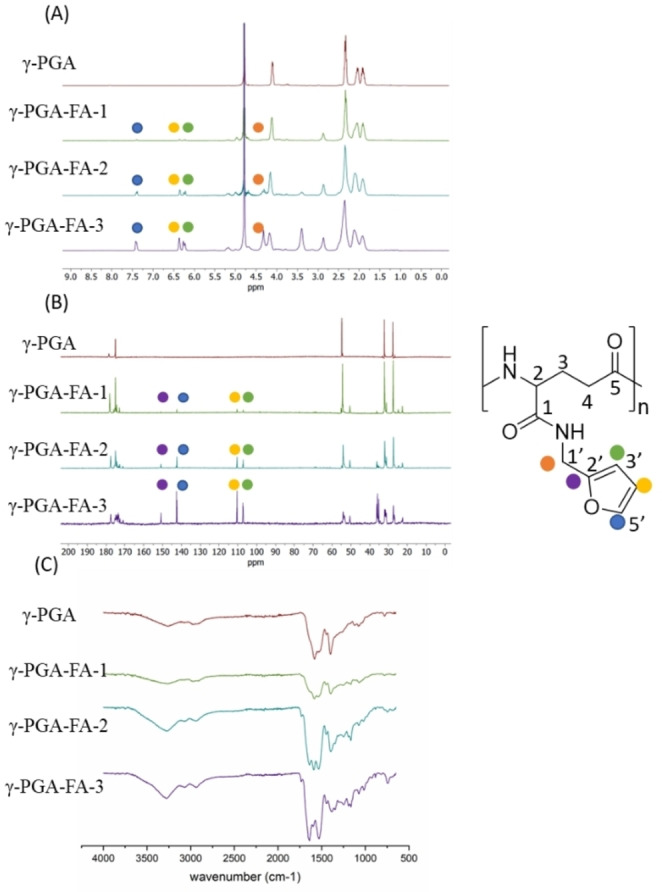
^1^H‐NMR (A), ^13^C‐NMR (B) and FT‐IR (C) spectra of γ‐PGA, γ‐PGA‐FA‐**1**, γ‐PGA‐FA‐**2** and γ‐PGA‐FA‐**3**.

The functionalization degree of each sample was calculated from the relative area ratio of ^1^H‐NMR peak integral at 6.2, 6.3 and 7.4 ppm of furan‐H (H‐3’,4’,5’) vs. the γ‐PGA CHNH_2_ peak (H‐2) at 4.1 ppm and/or CH_2_−C−CN peak at 2.3 ppm (H‐3) (Figure 2A).

FT‐IR analysis confirmed the functionalization with furan, by comparison between the derivatized and the untreated γ‐PGA (Figure 2C). The spectrum of the starting material shows the signals of C=O stretch and N−H bend of secondary amide at 1581 and 1537 cm^−1^, while the signals of sp^3^ C−H bend is at 1401 cm^−1^. γ‐PGA‐FA samples are characterized by different signals due to the introduction of a new moiety. In particular, the peaks corresponding to the sp^2^ C−H bend (cis alkene), C=C bend and C−O−C stretch of furan ring are detectable respectively at 745, 884 and 1016 cm^−1^. Furthermore, the peak of amide N−H bend and C=C stretch are visible respectively at 1533 and 1641 cm^−1^.

## Synthesis and characterization of γ‐PGA‐Ex‐4 conjugates

**Figure 3:**
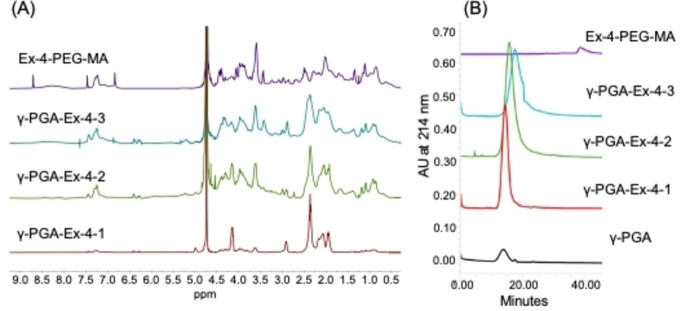
^1^H‐NMR (A) and SEC‐HPLC at 214 nm (B) of γ‐PGA‐Exendin‐4 conjugates: γ‐PGA‐Ex‐4‐**1**, γ‐PGA‐Ex‐4‐**2**, γ‐PGA‐Ex‐4‐**3**, Ex‐4‐PEG‐MA and the unmodified PGA (B).

γ‐PGA‐FA‐**1**, γ‐PGA‐FA‐**2** and γ‐PGA‐FA‐**3** were employed to generate the γ‐PGA‐Exendin‐4 conjugates (γ‐PGA‐Ex‐4‐**1**, γ‐PGA‐Ex‐4‐**2** and γ‐PGA‐Ex‐4‐**3**) by Diels‐Alder reaction with Ex‐4 functionalized with PEG_4_‐maleimide at Lys‐27 (Ex4‐PEG‐MA). The reactions were performed in PBS buffer 0.01 м at pH 7.5. The purified products were analyzed by ^1^H‐NMR and SEC‐HPLC (Figure 3).

The derivatization of γ‐PGA with Ex‐4 was evaluated by ^1^H‐NMR analysis, considering chemical shifts and integrals of both the γ‐PGA and Ex‐4, with particular focus on the chemical moieties directly involved in the conjugation, the double bonds of furan and the maleimide. The complete disappearance of the maleimide‐related peak at 6.8 ppm and the absence of the signals at 6.3 and 5.9 ppm, peculiar of maleimide ring opening, indicate the complete conjugation of Ex‐4. The degree of functionalization was calculated comparing the integrals of the protons of γ‐PGA (H‐4), at 2.4–2.3 ppm, and the signals of the aromatic residues of conjugated Ex‐4 (histidine, phenylalanine and tryptophan) at 7.4–6.9 ppm.^[84]^ The signals overlapping was considered in the integration. In detail, H‐5’ signal of unreacted furan residues was subtracted in the integration of the aromatic signals of Ex‐4, and the signals at 2.4‐2.3 ppm due to the Ex‐4 amino acid chains were subtracted in the integration of the H‐4 signal of γ‐PGA. Signals at 6.4 and 6.3 ppm reveals the presence of residual unreacted furan moieties, that can be exploited for further functionalization with additional species such as different epitopes or detecting agents, as reported in Supporting Information (Figure S13).

In SEC‐HPLC analyses, the elution of γ‐PGA‐Ex‐4 compounds, as well as γ‐PGA and Ex4‐PEG‐MA as controls, was monitored at 214 and 280 nm. The derivatized polymers experience a delay in the elution with respect to γ‐PGA, presumably due to the conformation change caused by the peptide conjugation. The protein content was determined from the ratio between the area of the peak at 280 nm, absent in the starting γ‐PGA‐FA, and the peak at 214 nm (Table 2). Furthermore, SEC‐HPLC confirmed the absence of free Ex4‐PEG‐MA in the sample.

**Table 2:** Characterization of γ‐PGA‐Ex‐4 conjugates: degree of functionalization and efficacy of γ‐PGA‐Ex‐4 conjugates measured by SEC‐HPLC, antibody and cell‐based assays.

Sample	^1^H‐NMR	SEC‐HPLC	Antibody‐based assay	Cell‐based affinity assay
	%DoF	peak area at 280 nm/214 nm	average number of Ex‐4/PGA molecule	relative affinity
γ‐PGA‐Ex‐4‐1	1.0 %	0.8 %	3	0.12
γ‐PGA‐Ex‐4‐2	6.0 %	3.3 %	46	0.61
γ‐PGA‐Ex‐4‐3	8.2 %	4.2 %	106	2.27

Antibody‐based assay was used to determine the number of Ex‐4 molecules conjugated to γ‐PGA, employing Alexa 488 polyclonal anti‐Ex4 antibody. In detail, Ex4‐conjugated polymers compete with Ex4‐coated beads (avidin‐coated beads conjugated with Ex4‐biotin) for the fluorescent anti‐Ex‐4 antibodies. The quantification of antibodies bound to the polymers was determined in a competitive flow cytometric assay. The calibration using known amounts of free Ex‐4 allowed to determine the average number of Ex‐4 molecules/γ‐PGA molecules. The results are in good correlation the data obtained by SEC‐HPLC and ^1^H‐NMR (Table 2).

## Affinity tests of γ‐PGA‐Ex‐4 conjugates with GLP‐1R

The binding ability of γ‐PGA‐Ex‐4 compounds to GLP‐1R expressed on β‐cells surface was examined in a competitive flow cytometric assay. This receptor binding assay was developed based on similar, receptor targeting studies, of Ex‐4 targeting^[85]^ and folate targeting.^[86]^ The relative competition capability to the target receptor, compared to Ex4‐PEG‐MA, was evaluated among γ‐PGA‐Ex‐4 conjugates at different degrees of functionalization. The obtained values were normalized with respect to the degree of functionalization of each bioconjugate, so that the comparison is not affected by the different amount of Ex‐4 molecules on each polymeric chain. GLP‐1R positive Beta‐TC‐6 cells were co‐incubated with fluorescein‐labelled Ex‐4 and the different Ex‐4 formulations, γ‐PGA‐Ex‐4‐1, γ‐PGA‐Ex‐4‐2, γ‐PGA‐Ex‐4‐3, and Ex4‐PEG‐MA or Ex‐4 as references.

**Figure 4:**
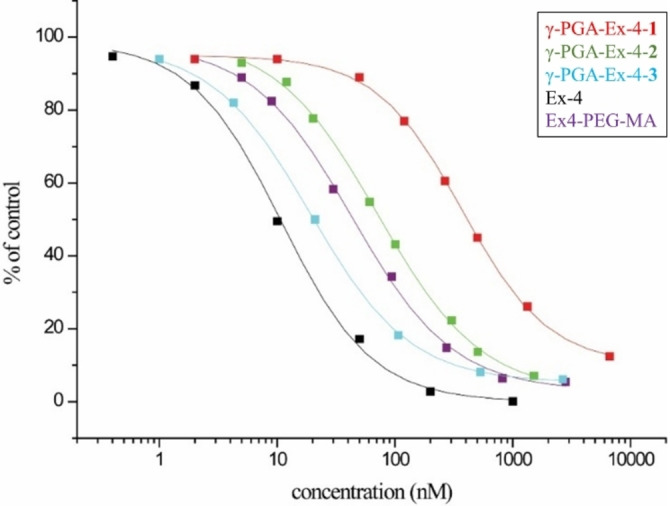
Binding ability of multivalent γ‐PGA‐Ex‐4 conjugates (γ‐PGA‐Ex‐4‐**1**, γ‐PGA‐Ex‐4‐**2**, γ‐PGA‐Ex‐4‐**3**) to beta cells compared to monovalent Ex‐4 and Ex4‐PEG‐MA peptides. Each compound is plotted against its molarity that in case of polymers is calculated considering an average *Mw*.

The results are plotted on Figure 4 in proportion to fluorescence intensity of cells incubated with fluorescent Ex‐4 alone as control. Ex‐4‐PEG‐MA, beside the derivatization, is still capable to bind GLP‐1R. The receptor binding ability of a single Ex‐4 molecule conjugated to γ‐PGA is necessarily reduced due to steric hindrance of the big cargo and the reduced degree of freedom and mobility. This justifies the lower relative affinity (RA) of γ‐PGA‐Ex‐4‐1 and 2. On the contrary, the higher number of Ex‐4 molecules on the γ‐PGA‐Ex‐4‐3 resulted in significantly higher affinity towards GLP‐1R positive β‐cells, double if compared to Ex4‐PEG‐MA. This result indicates the capability of γ‐PGA‐Ex‐4 conjugates to target β‐cells and the presence of a cluster effect, opening the way to multivalent Ex‐4 compounds with improved therapeutic efficacy.

## Conclusion

Conjugation of Ex‐4, an approved drug for diabetes type‐2 with γ‐PGA, a biocompatible and biodegradable FDA approved polymer, resulted in multivalent adducts with high affinity for GLP‐1R. The conjugation of Ex‐4 to γ‐PGA assures two key progresses: an increased affinity for GLP‐1R for the more intensively functionalized γ‐PGA‐Ex‐4 conjugate, and an increased stability due to the polymeric component. The γ‐PGA‐Ex‐4 conjugate presenting an average of 120 conjugated Ex‐4 molecules showed a double affinity towards GLP‐1R with respect to Ex4‐PEG‐MA. These results open the way to new improved therapeutic for diabetes and pancreas tumors.

## Experimental Section

**Materials**: Ex‐4‐PEG_4_‐Maleimide with the sequence HGEGTFTSDLSKQMEEE‐AVRLFIEWLys(PEG4‐Mal)KNGGPSSGAPPP S‐NH2 was purchased from Caslo ApS (Denmark) as freeze‐dried. Poly‐γ‐glutamic acid (γ‐PGA), with a molecular weight of 100 kDa, was gently granted by BBS Nanotechnology Ltd. (Hungary). *N*‐(3‐dimethylaminopropyl)‐*N*’‐ethylcarbodiimide hydrochloride (EDC, ≥98 %), 2‐(*N*‐morpholino) ethanesulfonic acid hydrate (MES, ≥99.5 %), 2‐(aminomethyl)furan (FA), phosphate buffered saline (PBS, tablets) and deuterium oxide were purchased by Sigma‐Aldrich (Germany). Polymers purification was performed using cellulose membranes (14,000 Da cut‐off) or Vivaspin tubes with a molecular weight cut‐off of 30 kDa, MilliQ water and a solution 0.1 м NaCl in MilliQ. The polymers have been freeze‐dried using a ALPHA 1–2 LO freeze‐dryer.

**γ‐PGA functionalization with furan (γ‐PGA‐FA‐1, γ‐PGA‐FA‐2 and γ‐PGA‐FA‐3)**: γ‐PGA (100 mg, 0.69 mmol) were solubilized in MES buffer solution 0.1 м pH=5.5 (10 mL). EDC/NHS (0.5, 1 or 2 equivalent units each) were added as powder and the solution stirred at room temperature for 30 minutes. Following, FA was added dropwise (0.25, 0.5 or 2 equivalents in different experiments) and left reacting for 24 hours. The solution was dialyzed in a cellulose membrane dialyzing tube with a molecular weight cut‐off of 14 kDa, against NaCl 0.1 м and then MilliQ water, for 48 hours. Finally, the solution was freeze‐dried, giving the products γ‐PGA‐FA‐1, γ‐PGA‐FA‐2 and γ‐PGA‐FA‐3 respectively as powder.

**Conjugation of γ‐PGA‐FA with Ex‐4. (γ‐PGA‐Ex‐4‐1, γ‐PGA‐Ex‐4‐2 and γ‐PGA‐Ex‐4‐3**): γ‐PGA‐FA‐**1** (2 % of functionalization, 10 mg), solubilized in PBS 0.01 м pH=7.5 (1 mL) was treated with 1 eq. of Ex4‐PEG‐MA solubilized in PBS pH=7.5 (1, 2 and 4 mL). After stirred overnight at room temperature, the product was isolated with PBS in Amicon® Ultra‐15 Centrifugal Filter Units (MWCO 30 kDa) at 5,000 g for 10 minutes 15 times, and finally freeze‐dried. γ‐PGA‐FA‐**2** (8 % of functionalization) and γ‐PGA‐FA‐**3** (16 % of functionalization) were separately treated with the same protocol, with 1 eq of Ex4‐PEG‐MA solubilized respectively 2 and 4 mL of PBS pH=7.5.

**NMR analysis**: All the analyzed samples were dissolved in D2O‐d2 (99,98 % deuterated; few mg in 0,6 ml of D2O). ^1^H‐NMR spectra were acquired using a *Bruker BioSpin* FT‐NMR ***Avance*** ^**TM**^ ***I 600*** (^1^H frequency=600 MHz) equipped with a superconducting ultrashield magnet of 14,1 Tesla, with pulse field gradient module (Z axis) and a tunable 5 mm *reverse broadband BBI* probe NMR, with high sensitivity on ^1^H nucleus and on a Bruker BioSpin FT‐NMR Avance^TM^ 500 (^1^H frequency=500 MHz) equipped with a superconducting ultrashield magnet of 11.7 Tesla, with pulse field gradient module (Z axis) and a tunable 5 mm direct QNP probe (13C, 31P, 19F and 1H nuclei), to increase sensitivity on 13C nucleus., setting the pulse angle at 90° and the relaxation delay at 5 seconds, while the number of scans was varied between 160 and 320, depending on the signal‐to‐noise ratio.

**FI‐IR analysis**: FT‐IR spectra have been performed with a spectrometer PerkinElmer Spectrum 100 equipped with a Universal ATR (UATR). The range of acquisition is 4000–650 cm^−1^ with a resolution of 2 cm^−1^, while the absorbances of the samples and backgrounds were measured using 30 scans each.

**HPLC‐SEC analysis**: Size exclusion chromatography analysis was carried out with a Waters e2695 Separations Module (Waters Co., Milford, MA, USA) HPLC system using an Ultrahydrogel 1000 column (Waters, 7.8×300 mm,12 μm) equipped with a PDA detector (Waters 2998 PDA detector). The flow rate was set to 0.60 mL/min using gradient elution and column was maintained at 303 K. The eluent was 100 % PBS buffer which pH was adjusted to 7.4 and changed to 90 % PBS: 10 % Methanol from the 35^th^ minute.

**Determination of average number of Ex4 molecules conjugated to γ‐PGA molecules (antibody‐based assay)**: γ‐PGA‐Ex‐4 compounds were analyzed based on their recognition by Alexa Fluor 488 conjugated polyclonal anti‐Ex4 antibody (Bioss Inc.). The number of antibodies bound to the polymers was determined in a competitive flow cytometric assay, Ex4‐conjugated polymers competed with Ex4‐coated beads for the A488‐anti‐Ex‐4.

Preparation of Ex‐4 coated beads: avidin‐coated beads (d=0.86 μm, Spherotech Inc) were mixed to six times excess of Ex4‐biotin (AnaSpec Inc) in PBS‐0.1 % BSA at RT, after 0.5 h the beads were washed two times with 1 mL PBS‐0.1 % BSA (13 000 g, 5 min).

The competitive test: the Ex‐4‐containing samples in different concentration were incubated with 3×10^6^ Ex‐4 coated beads in presence of 5 μg/mL A488 conjugated anti‐Ex‐4 in 60 μl PBS‐0.1 %BSA. After 1 h incubation at 310 K the beads were washed with 1 mL PBS‐0.1 %BSA (13 000 g, 5 min) and the fluorescence of the beads was measured by flow cytometer (BD FACSCalibur). Flowing Software was used for data evaluation. The calibration of this assay with known amounts of free Ex‐4 allowed the determination of the average number of Ex‐4 molecules/γ‐PGA molecules.

**Affinity test (cell‐based assay)**: GLP‐1R positive Beta‐TC‐6 cells (ATCC) were dissociated using TrypLE™ Express Enzyme (Gibco).

5×10^5^ cells were incubated with 10 nм fluorescein‐labelled Ex‐4 (Eurogentec) alone or together with the tested compounds at 310 K in RPMI supplemented with 10 % FBS. After 0.5 h the cells were washed with ice cold PBS and fluorescence of the cells was measured by flow cytometer (BD FACSCalibur). Flowing Software was used for data evaluation. The binding ability of γ‐PGA‐Ex‐4 conjugates and peptides to the GLP‐1R positive cells was calculated based on the inhibition of the fluorescent Ex‐4 derived signal. The relative affinities were defined as the ratio of molar concentrations of Ex4‐PEG‐MA and the conjugated sample required to displace 50 % of fluorescent Ex‐4 bound to the GLP‐1R of beta cells:

$$relative\;affinity\;\left(RA\right)=\;\frac{{IC}_{50}\left(Ex-4-PEG-maleimide\right)}{{IC}_{50}\left(\gamma -PGA-Ex-4\right)}$$

## Conflict of interest

The authors declare no conflict of interest.

1

## Supporting information

As a service to our authors and readers, this journal provides supporting information supplied by the authors. Such materials are peer reviewed and may be re‐organized for online delivery, but are not copy‐edited or typeset. Technical support issues arising from supporting information (other than missing files) should be addressed to the authors.

Supporting Information

## Data Availability

The data that support the findings of this study are available in the supplementary material of this article.
