# Supplementary material for: Multivalent γ‐PGA‐Exendin‐4 Conjugates to Target Pancreatic β‐Cells
Source: Chembiochem. 2022 Jul 13;23(17):e202200196. doi: 10.1002/cbic.202200196 (PMC9542156; doi:10.1002/cbic.202200196)

ChemBioChem

Supporting Information

Multivalent γ-PGA-Exendin-4 Conjugates to Target Pancreatic β-Cells

Lorenzo Rossi, Krisztina Kerekes, Judit Kovács-Kocsi, Zoltán Körhegyi, Magdolna Bodnár, Erika Fazekas, Eszter Prépost, Cataldo Pignatelli, Enrico Caneva, Francesco Nicotra, and Laura Russo*

Contents

Supporting Section 1. NMR investigation of γ-PGA-FA-1

Reference sample "LRN35-56", with 2% of theoretical Furan coordination, was performed with a Bruker BioSpin FT-NMR Avance™ I 600 (1H frequency = 600 MHz) equipped with a superconducting ultrashield magnet of 14,1 Tesla, with pulse field gradient module (Z axis) and a tunable 5 mm reverse broadband BBI probe NMR, with high sensitivity on ^{1}H nucleus; other experiments were performed on a Bruker BioSpin FT-NMR Avance™ 500 (1H frequency = 500 MHz) equipped with a superconducting ultrashield magnet of 11.7 Tesla, with pulse field gradient module (Z axis) and a tunable 5 mm direct QNP probe (13C, 31P, 19F and 1H nuclei), to increase sensitivity on 13C nucleus. ^{1}H spectra were acquired, not spinning, at T = 303 K (as in all subsequent experiments), with the presaturation of H_2O signal (zgpr), using following parameters: spectral width (sw) = 6613.76 Hz, acquisition time (at) = 2.47 s, time domain (td) = 32k, relaxation delay (d1) = 5 s, number of scans (ns) = 320; ^{13}C NMR inverse gated zgig, ^{1}H all decoupled spectrum, was run with: sw = 220,79 ppm, at = 0.49 s, td = 32K, d1 = 2 s, ns = 7000; DQF-COSY: d1 = 1 s, at = 0.34 s and 256 increments in t1; HSQC: d1 = 1 s, at = 0.34 s, 128 increments in t1) and HMBC (d1 = 1.5 s, at = 0.17 s, 128 increments in t1, long range JC-H = 8 Hz).

DOSY experiments (Diffusion Ordered Spectroscopy), also called PFG-NMR (Pulsed Field Gradient - NMR), were performed with bipolar gradient pulses, for diffusion, using 2 spoil gradients[39], with a field gradient power, in the z-direction, of 53.5 G cm_1. The length of the magnetic field pulse gradient (δ) was optimized, in correlation with different diffusion time (Δ) values, in order to obtain a 2 to 5% medium residual signal on the whole spectra, in correspondence to the application of 95% of the maximum gradient strength. The optimized value of δ was of 2,5

ms, while Δ was of 800 ms (needed for the high dimension of the studied molecules); the pulse gradients (g) were incremented from 2 to 95% of the maximum gradient strength, in a linear ramp mode. DOSY experiment has been acquired with: d1 = 2 s, at = 2,72 s, 64 increments in t1 (number of gradient increments in the second dimension) and the eddy current delay (Te) was set to 5 ms.

Supporting Figures S1-S13

Figure S1. ^{1}H-NMR – γ-PGA-FA-1 (degree of functionalization 2%)

^{1}H-NMR (deuterium oxide, solvent presaturation, 303K, BBI probe, 600 MHz): δ_H 7.41 (0.02 H, d, J= 14.88 Hz), 6.37 (0.02 H, s), 6.25 (0.02 H, d, J= 24.52 Hz), 4.97 (0.08 H, m), 4.31 (0.04 H, m), 4.13 (0.8 H, m), 2.87 (0.26 H, s), 2.33 (2 H, t, J= 8.28 Hz), 2.06 (1.25 H, m), 1.91 (0.84 H, m)

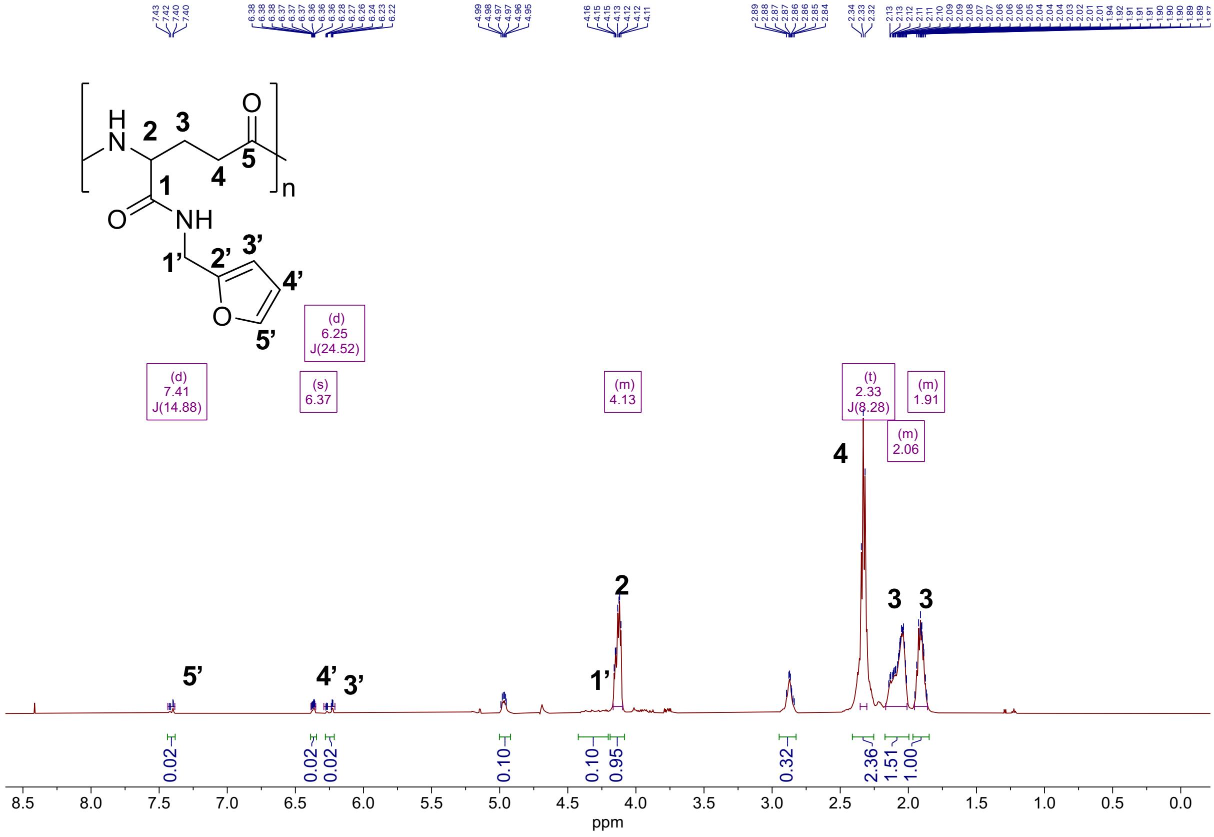

Figure S2. ^{1}H-NMR – γ-PGA-FA-2 (degree of functionalization 8%)

^{1}H-NMR (deuterium oxide, solvent presaturation, 303K, QNP probe, 500MHz): δ_H 7.45 (0.08 H, d, J= 11.21 Hz), 6.41 (0.08 H, s), 6.29 (0.09 H, d, J= 18.72 Hz), 5.22 (0.07 H, d), 4.97 (0.07 H, s), 4.36 (0.16 H, m), 4.16 (0.67 H, m), 3.45 (0.25H, m), 2.90 (0.3 H, s), 2.36 (2 H, t, J= 7.27 Hz), 2.09 (1.18 H, m), 1.95 (0.72 H, m)

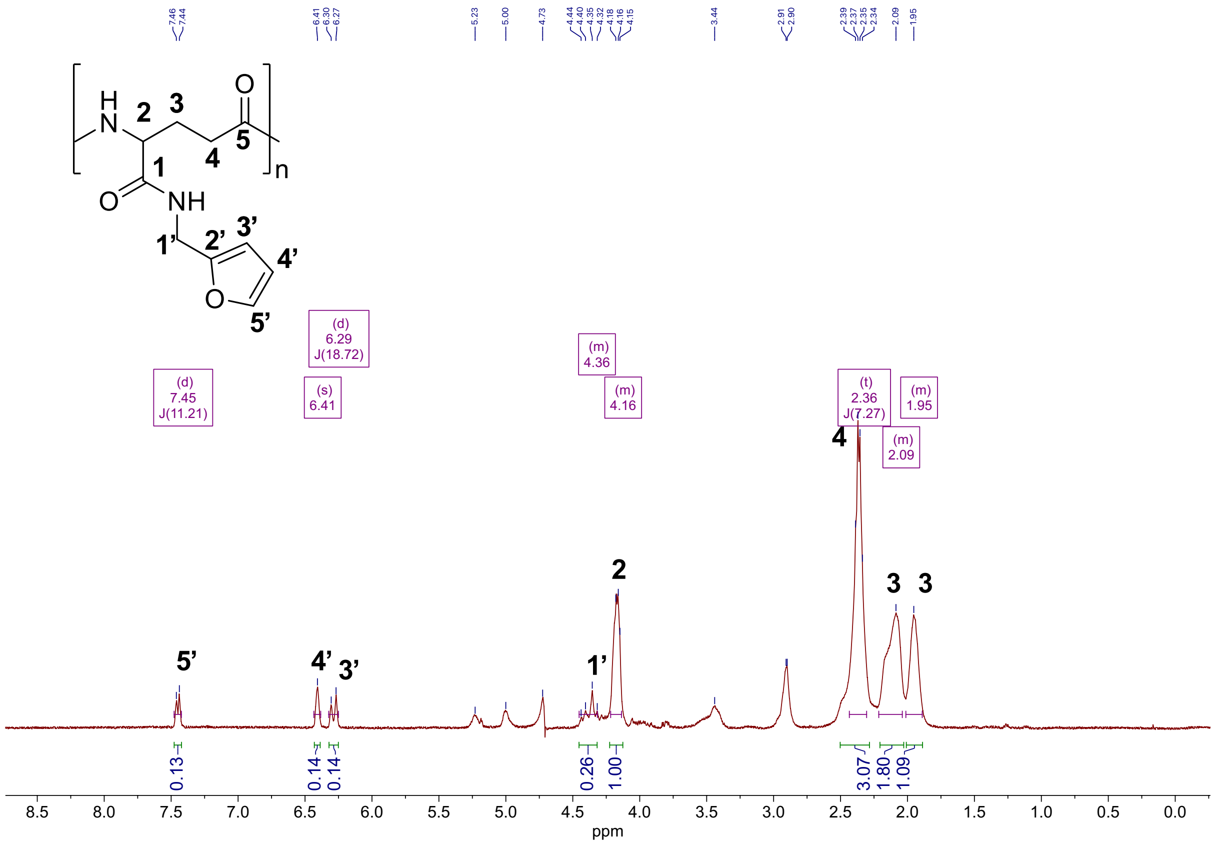

FIGURE S3. ^{1}H-NMR – γ-PGA-FA-3 (degree of functionalization 16%)

^{1}H-NMR (deuterium oxide, solvent presaturation, 303K, QNP probe, 500MHz): δ_H 7.44 (0.16 H, d, J= 10.09 Hz), 6.39 (0.17 H, d), 6.27 (0.17 H, d, J= 18.70 Hz), 5.22 (0.08 H, d), 4.34 (0.32 H, m), 4.18 (0.45 H, m), 3.45 (0.6 H, m), 2.90 (0.28 H, s), 2.36 (2 H, t, J= 19.6 Hz), 2.11 (0.88 H, m), 1.93 (0.5 H, m)

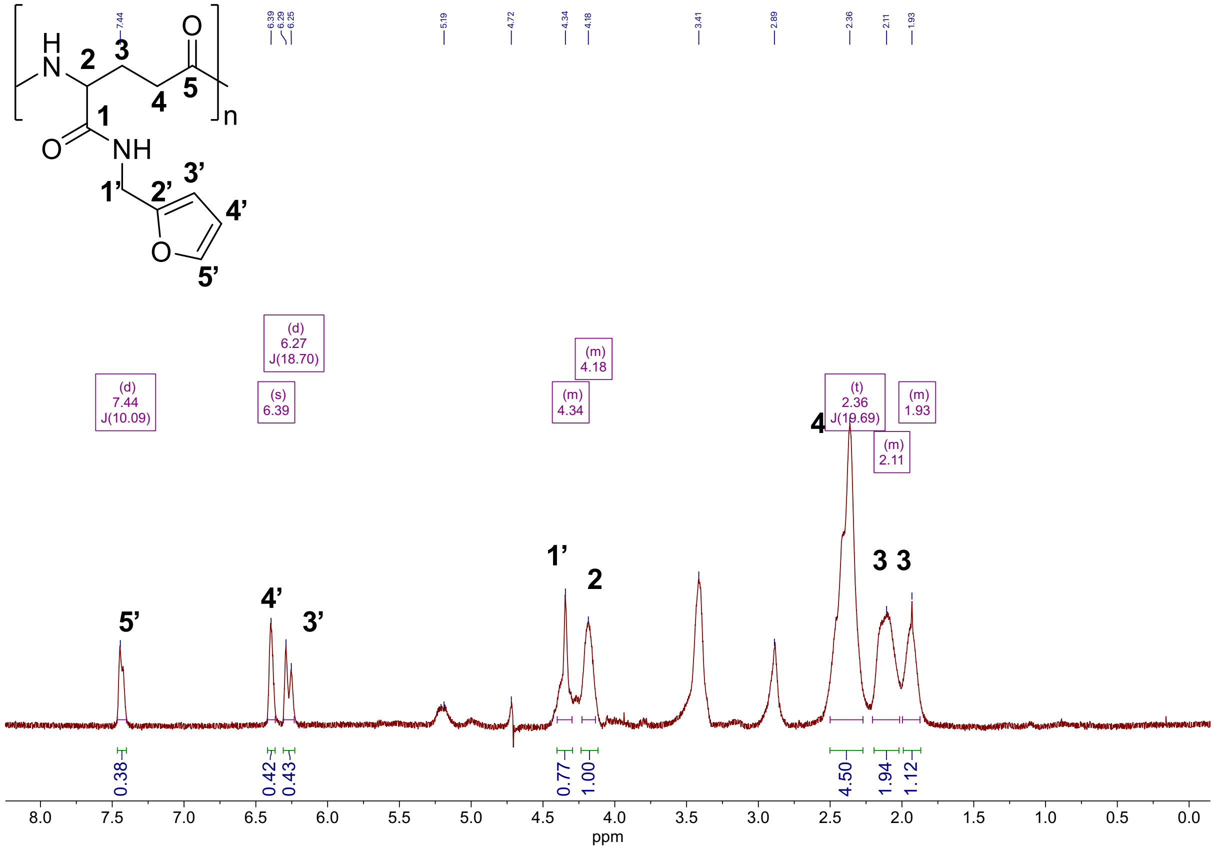

FIGURE S4. ^{1}H-^{1}H correlation evidence – γ-PGA-FA-1

^{1}H-COSY-gpdqf (deuterium oxide, 303K, BBI probe, 600 MHz)

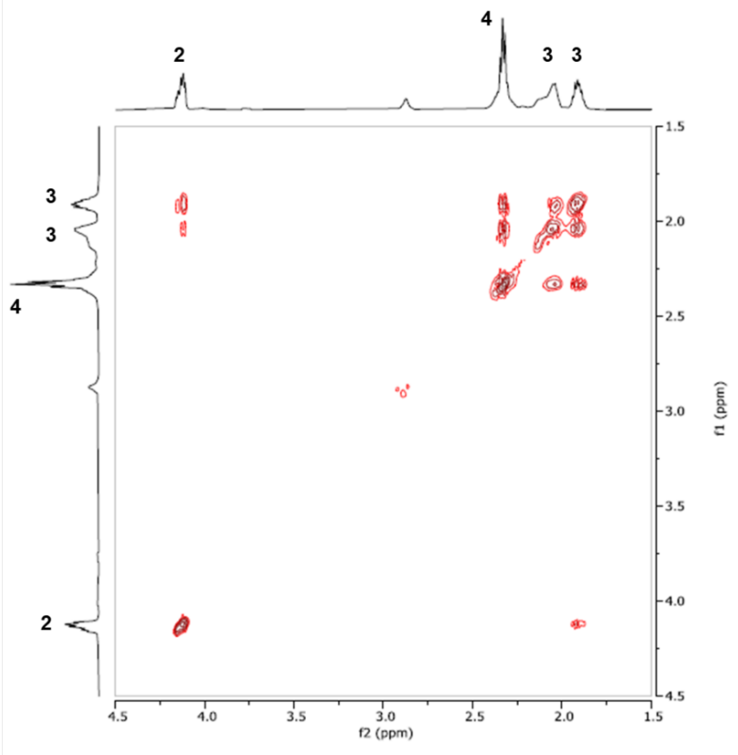

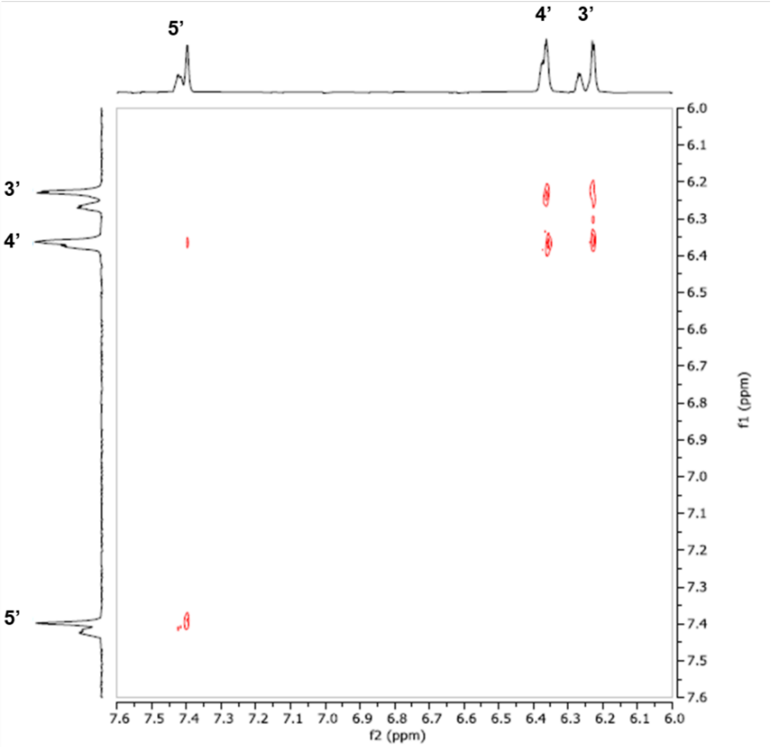

FIGURE S5. ^{1}H-^{13}C one bond correlation – γ-PGA-FA-1

^{1}H-^{13}C HSQCesgp (deuterium oxide, 303K, BBI probe, 600 MHz)

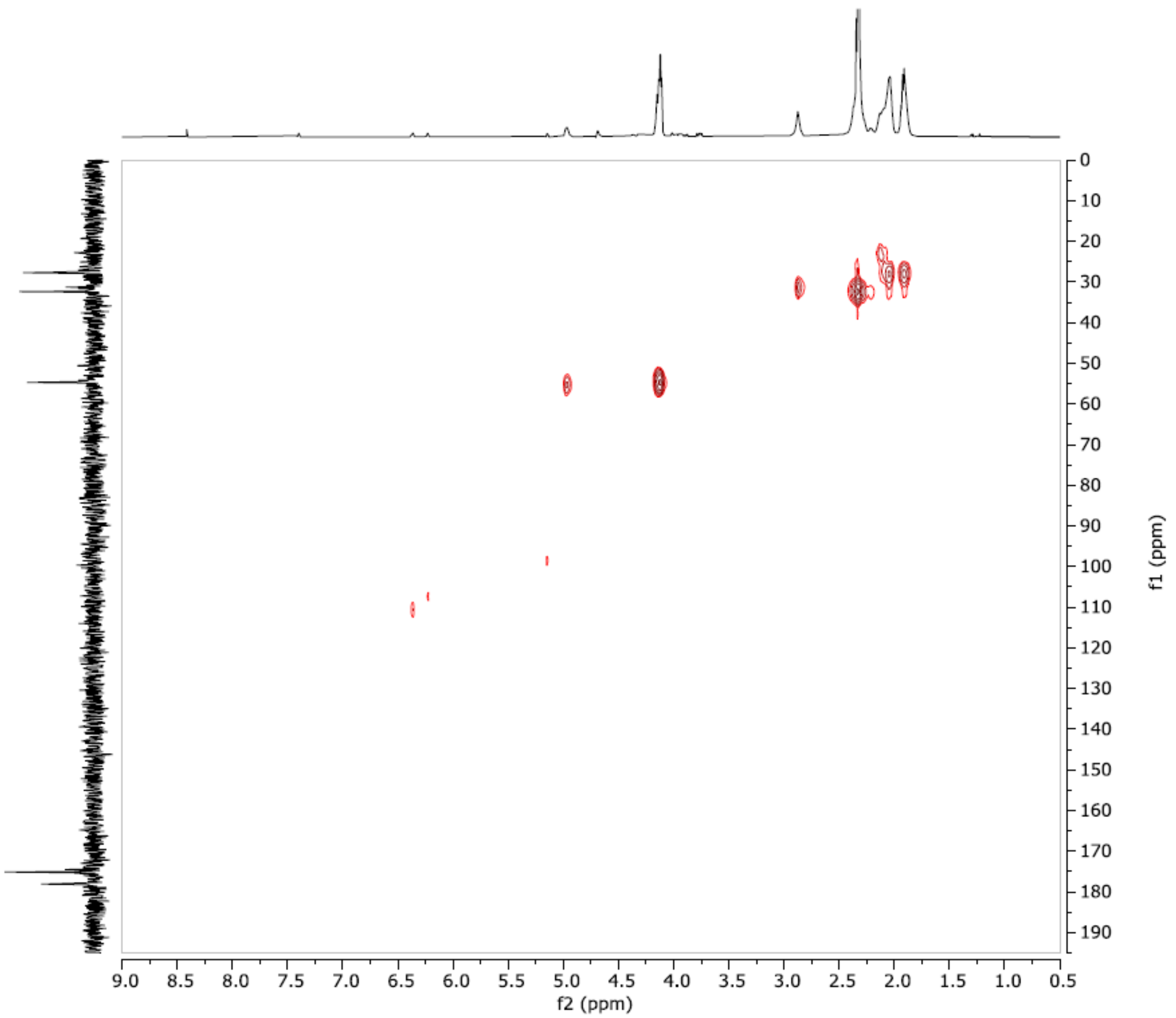

FIGURE S6. ^{1}H-^{13}C multiple bond correlation – γ-PGA-FA-1, in two different processing mode

^{1}H-^{13}C Long Range correlations - HMBC (deuterium oxide, 303K, BBI probe, 600 MHz)

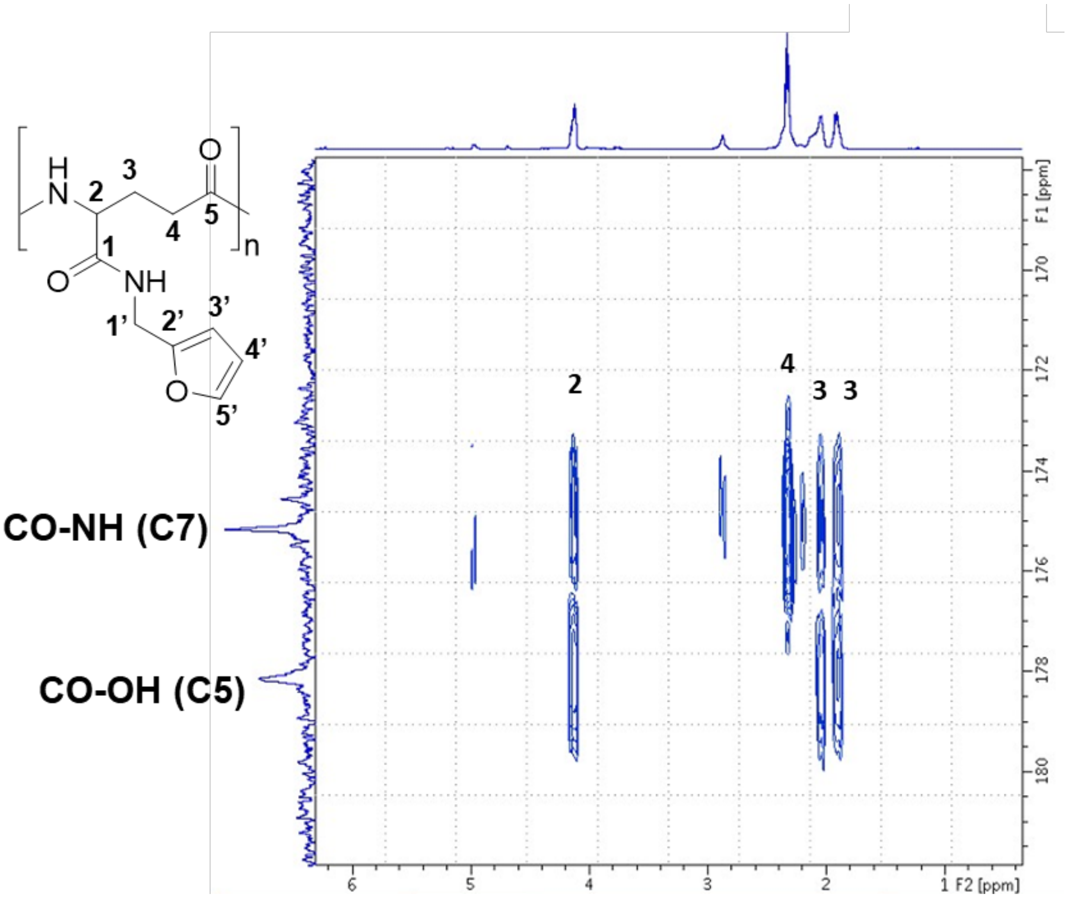

FIGURE S7. [1]H-DOSY – γ-PGA-FA-1

DOSY (deuterium oxide, p30=2.5ms; d20=800ms; d1=2s, 303K, BBI probe, 600 MHz)

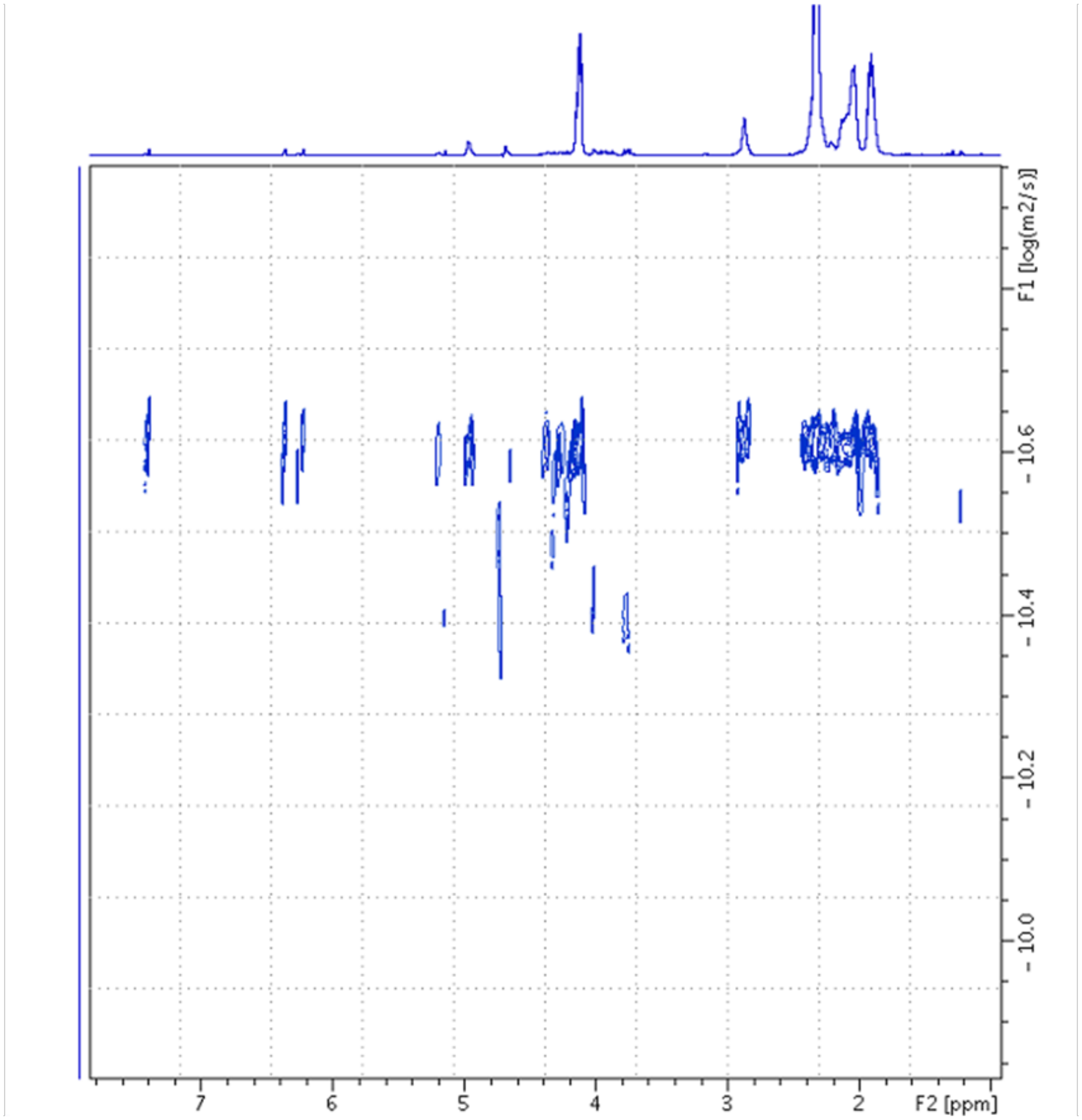

FIGURE S8. ^{1}H-NMR – Ex4-PEG-MA

^{1}H-NMR (deuterium oxide, 303K, 400 MHz): δ_H 7.40-6.90 (17 H, m), 6.83 (2H, s)

Integral of the peaks between 2.43 and 2.30 ppm was calculated with respect to the maleimide and aromatic residues signals. This information has been used for subsequent subtraction of aminoacidic chains of Ex-4 from PGA γNH H-4 peak in the calculation of the degree of functionalization of γ-PGA-Ex-4 conjugates.

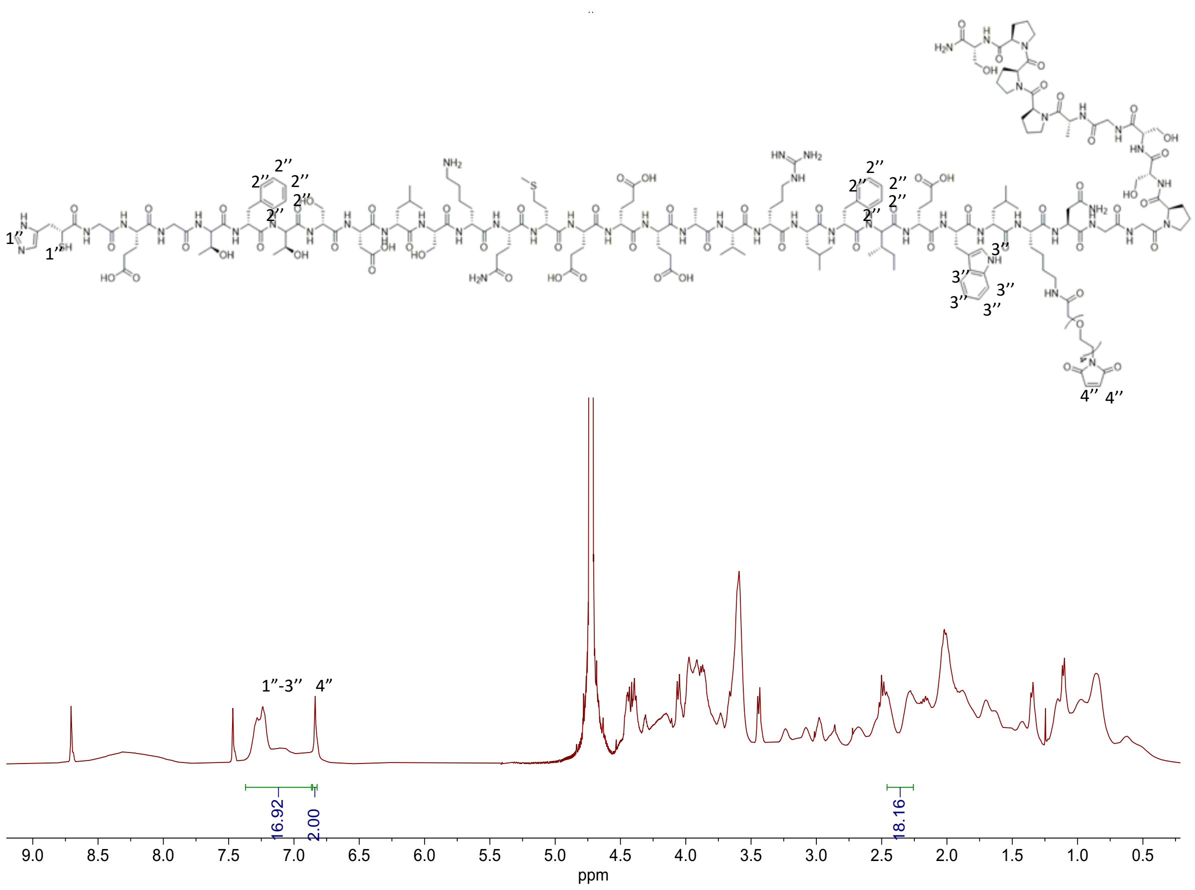

FIGURE S9. ^{1}H-NMR – a) comparison between Ex4-PEG-MA, γ-PGA-Ex-4-1, γ-PGA-Ex-4-2, γ-PGA-Ex-4-3 and Ex4-PEG-MA quenched, b) focus between 8.10 and 5.70 ppm

a)

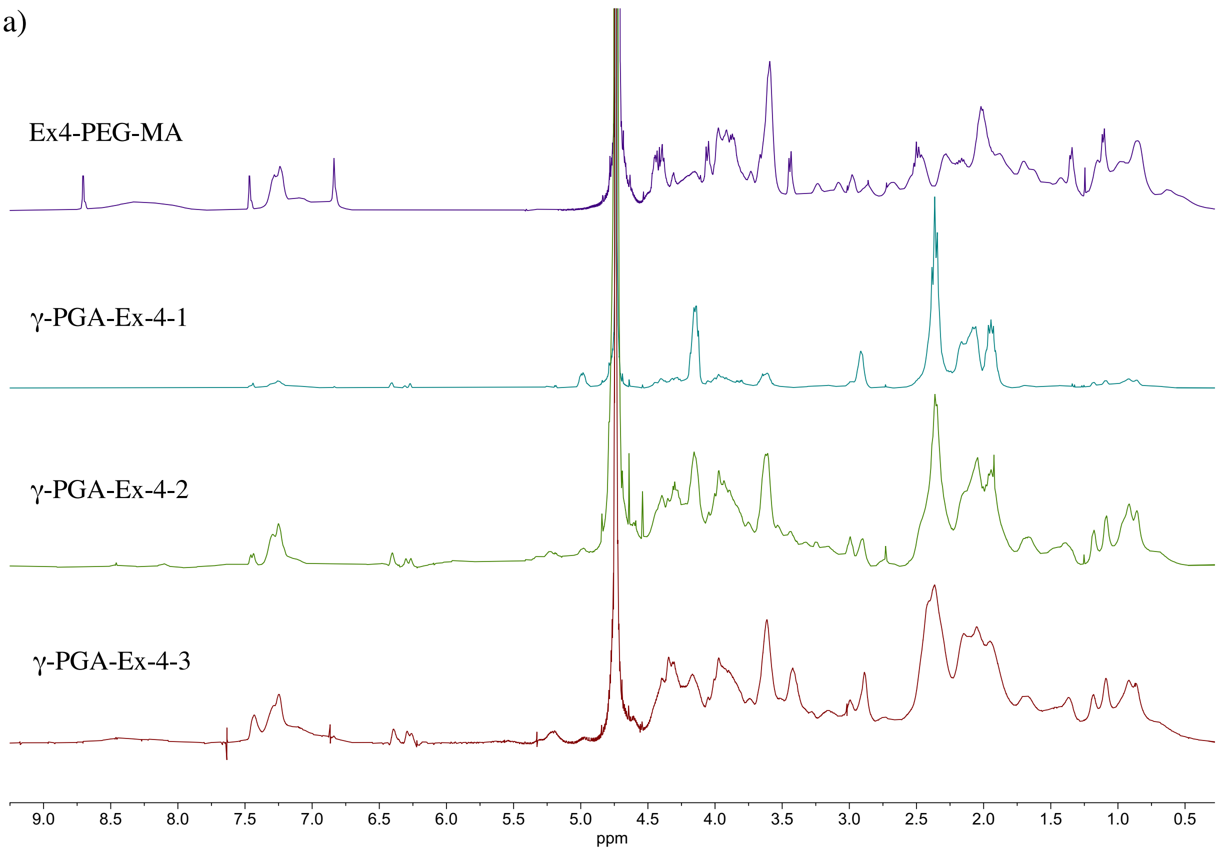

b)

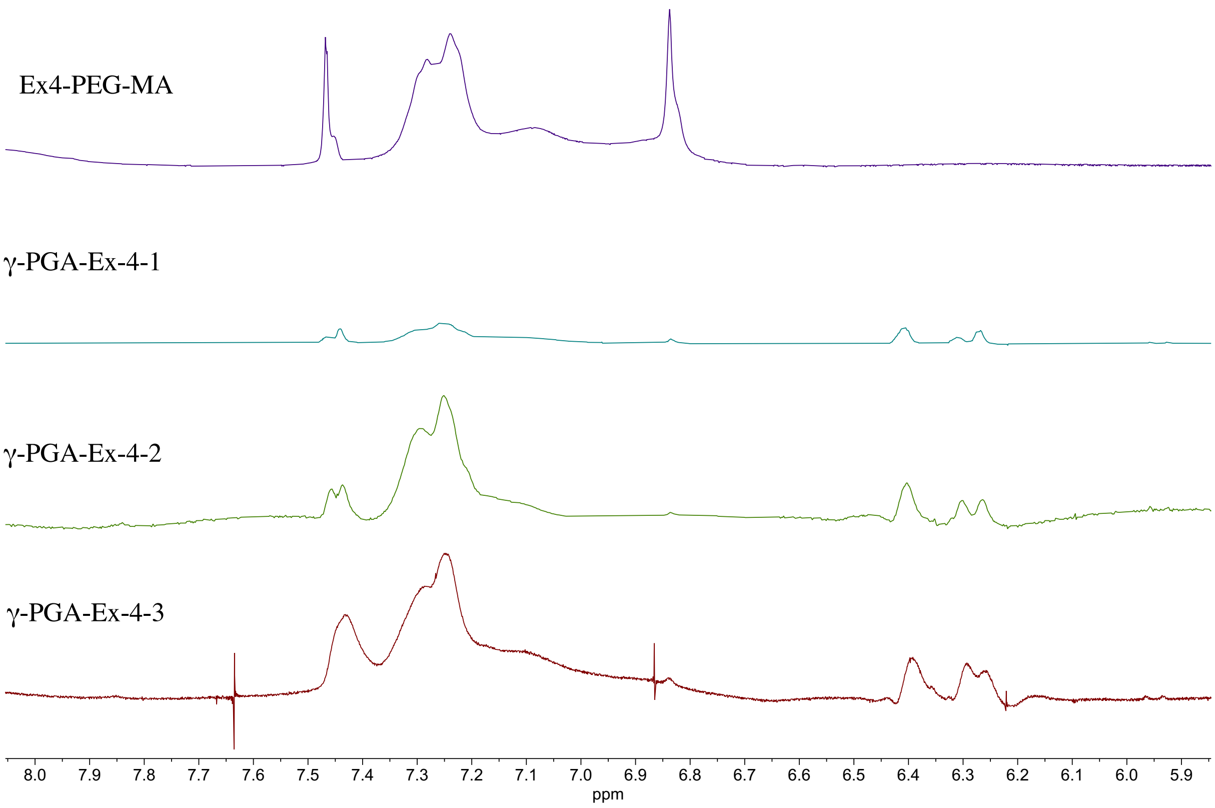
Ex4-PEG-MA
γ-PGA-Ex-4-1
γ-PGA-Ex-4-2
γ-PGA-Ex-4-3
8.0 7.9 7.8 7.7 7.6 7.5 7.4 7.3 7.2 7.1 7.0 6.9 6.8 6.7 6.6 6.5 6.4 6.3 6.2 6.1 6.0 5.9
ppm

FIGURE S10. ^{1}H-NMR – γ-PGA-Ex-4-1

^{1}H-NMR (deuterium oxide, 303K, 400 MHz): δ_H 7.40-6.90 (0.02 H, m), 6.41 (0.02H, s), 6.29 (0.02 H, d), 2.43-2.30 (2.17 H, t)

The degree of functionalization (1%) was estimated by comparison between the integrals of the peak of γNH H-4, from which the integral of aminoacidic chains of Ex-4 were subtracted, and the integral of H-1''-3'' of histidine, two phenylalanine and tryptophan residues of Ex-4, from which the integral of the signal related to unreacted furan was subtracted.

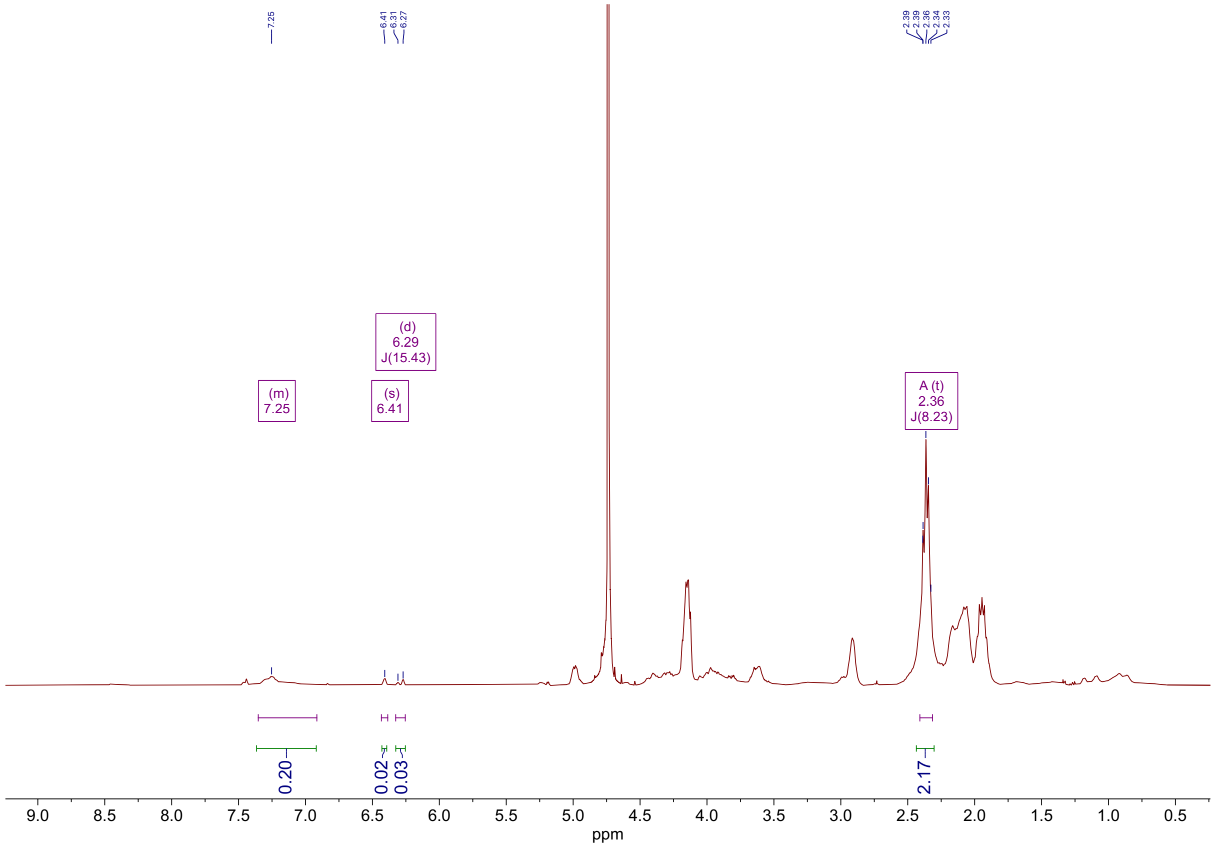

FIGURE S11. ^{1}H-NMR – γ-PGA-Ex-4-2

^{1}H-NMR (deuterium oxide, 303K, 400 MHz): δ_H 7.40-6.90 (1.08 H, m), 6.40 (0.06 H, s), 6.28 (0.06 H, d), 2.43-2.30 (2.9 H, m)

The degree of functionalization (6%) was estimated by comparison between the integrals of the peak of γNH H-4, from which the integral of aminoacidic chains of Ex-4 were subtracted, and the integral of H-1''-3'' of histidine, two phenylalanine and tryptophan residues of Ex-4, from which the integral of the signal related to unreacted furan was subtracted.

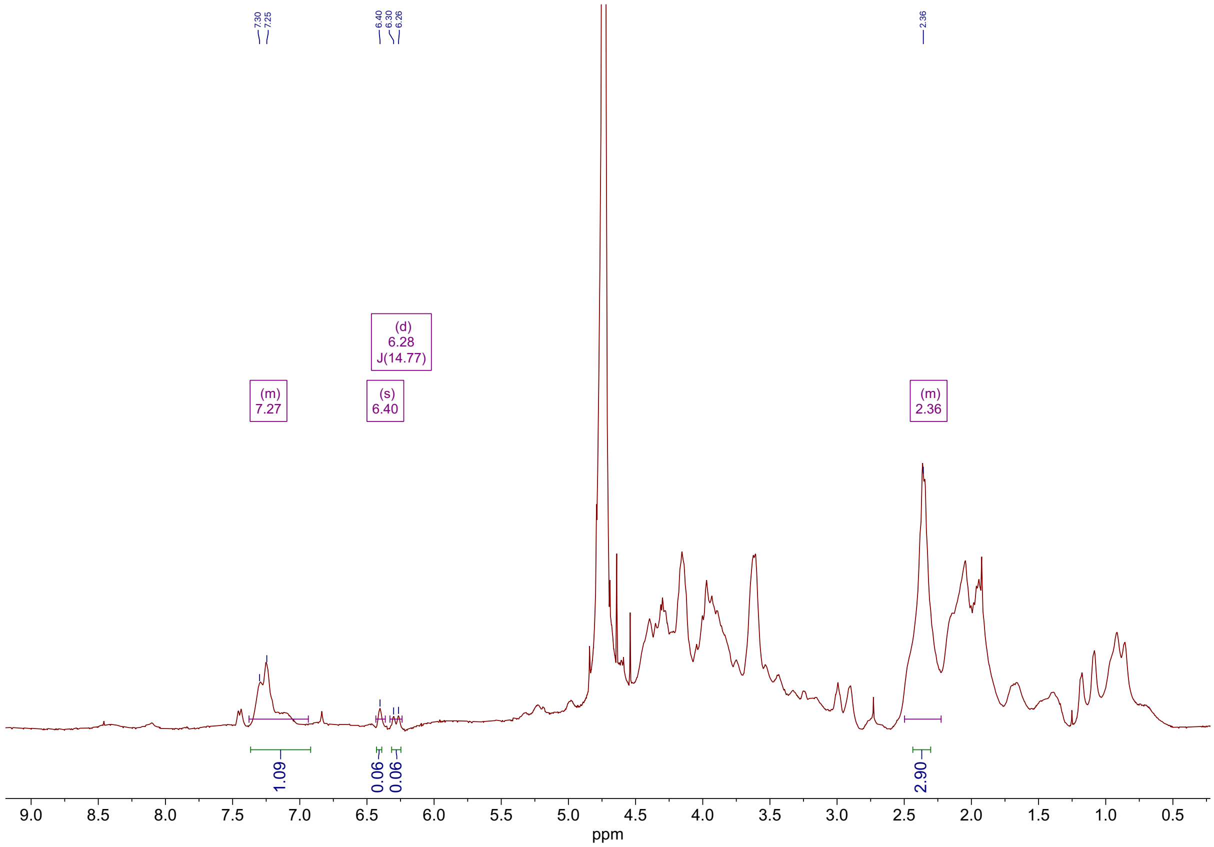

FIGURE S12. ^{1}H-NMR – γ-PGA-Ex-4-3

^{1}H-NMR (deuterium oxide, 303K, 400 MHz): δ_H 7.40-6.90 (1.5 H, m), 6.39 (0.1 H, s), 6.28 (0.1 H, d), 2.43-2.30 (3.44 H, m)

The degree of functionalization (8.2%) was estimated by comparison between the integrals of the peak of γNH H-4, from which the integral of aminoacidic chains of Ex-4 were subtracted, and the integral of H-1''-3'' of histidine, two phenylalanine and tryptophan residues of Ex-4, from which the integral of the signal related to unreacted furan was subtracted.

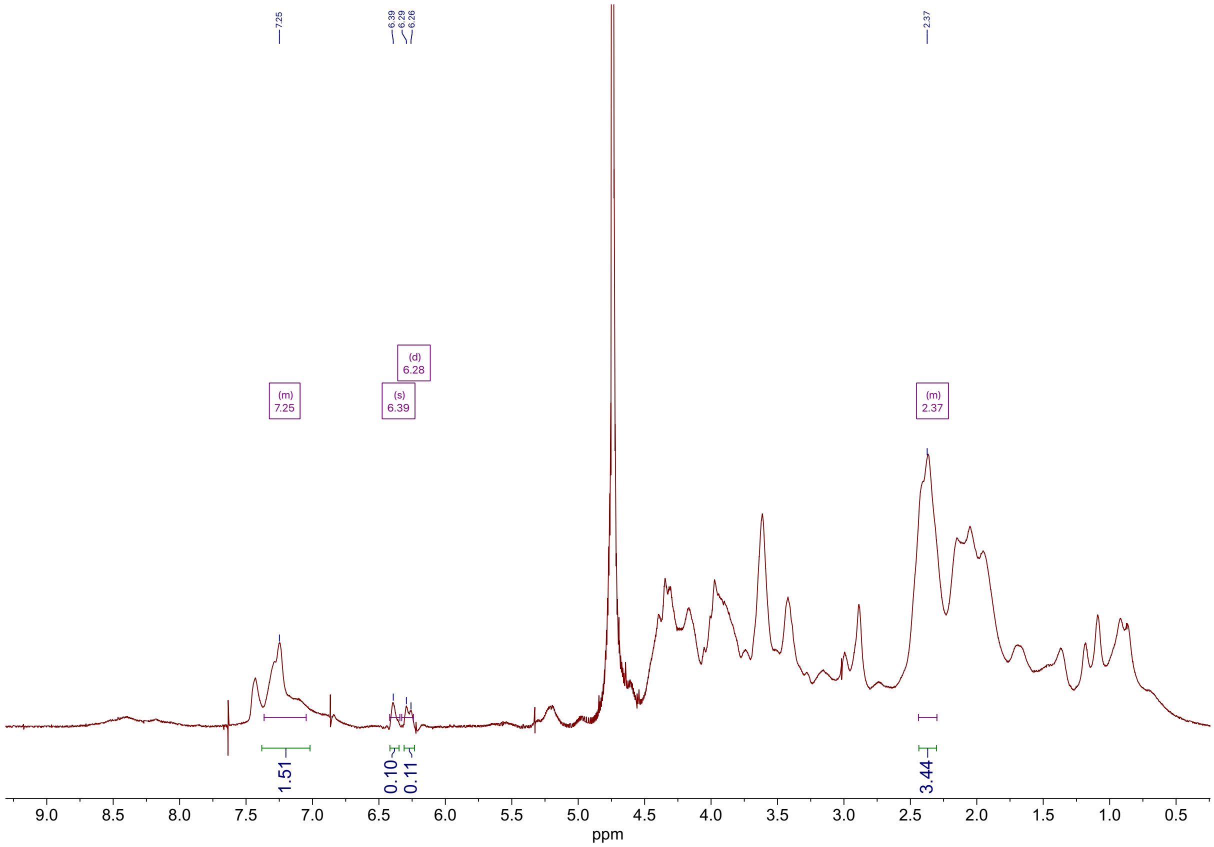

Figure S13. ^{1}H-NMR – γ-PGA-Ex-4-3 further functionalized with Ex-4-PEG-MA

γ-PGA-Ex-4-3 was studied by ^{1}H-NMR in presence of an excess of Ex-4-PEG-MA. After 30 minutes, the signal related to Ex-4-PEG-MA maleimide at 6.8 ppm decreases, confirming that the residual furan moieties of γ-PGA-Ex-4-3 can be further functionalized.

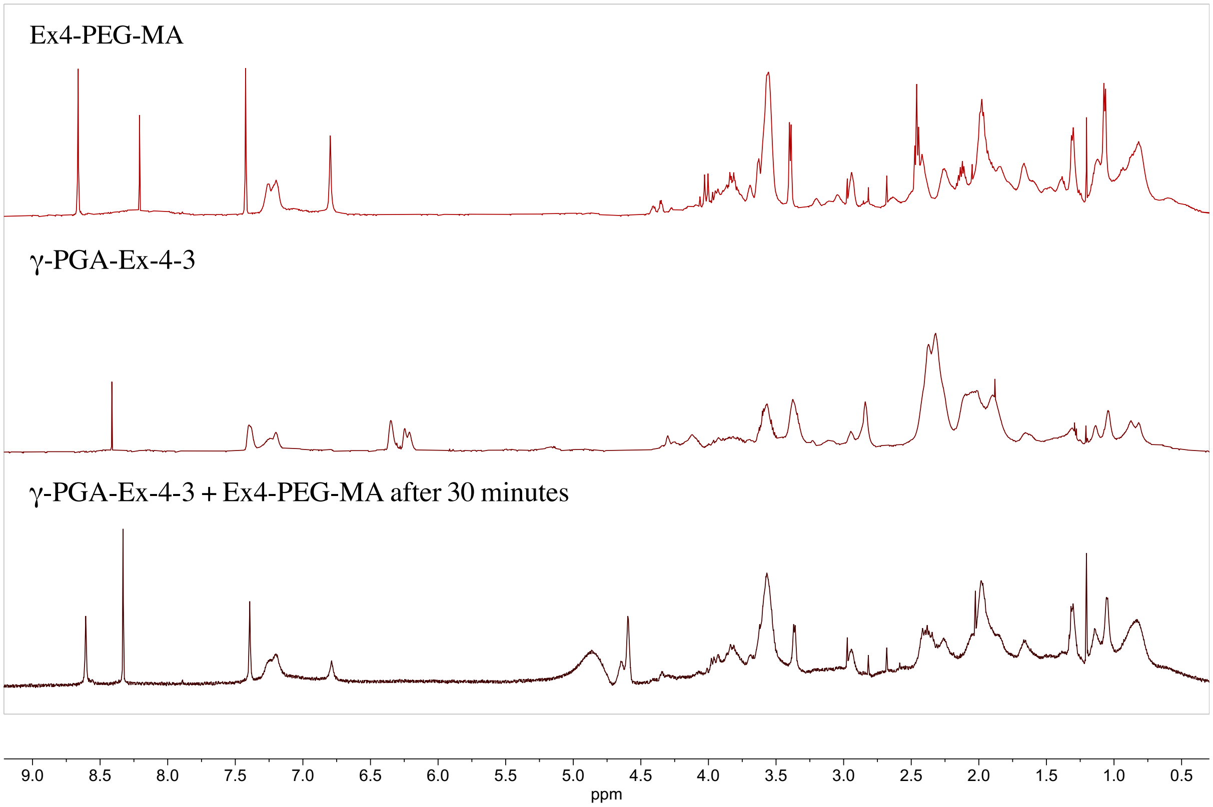

Supplement: Supplementary file 1 — Supporting Information [file CBIC-23-0-s001.pdf]
